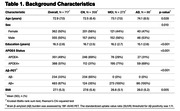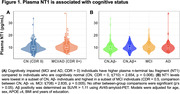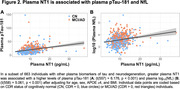# Plasma N‐terminal tau fragment is associated with cognitive status and AD biomarkers of tau and neurodegeneration in older adults

**DOI:** 10.1002/alz.093896

**Published:** 2025-01-09

**Authors:** Yiwen Rao, Beth L. Ostaszewski, Amirah K. Anderson, Zahra Shirzadi, Lei Liu, Hyun‐Sik Yang, Jasmeer P. Chhatwal, Dennis J. Selkoe, Stephanie A. Schultz, ADNI

**Affiliations:** ^1^ Department of Neurology, Brigham and Women's Hospital, Boston, MA USA; ^2^ Department of Neurology, Brigham and Women's Hospital, Harvard Medical School, Boston, MA USA; ^3^ Massachusetts General Hospital, Harvard Medical School, Boston, MA USA; ^4^ Ann Romney Center for Neurologic Diseases, Department of Neurology, Brigham and Women's Hospital, Harvard Medical School, Boston, MA USA; ^5^ Brigham and Women's Hospital, Boston, MA USA; ^6^ Alzheimer's Disease Neuroimaging Initiative, http://adni.loni.usc.edu/, CA USA

## Abstract

**Background:**

The emergence of blood‐based biomarkers offers a cost‐effective and less invasive alternative to established neuroimaging and cerebrospinal fluid biomarkers. Newly developed fluid biomarkers, including N‐terminal tau fragment (NT1), have shown promise for identifying individuals at risk for Alzheimer’s disease (AD). Evidence has shown NT1 may be more abundant than full‐length tau across the AD continuum and has high sensitivity and specificity to separate cognitively normal (CN) individuals from those with mild cognitive impaired (MCI) and AD in discovery and replication cohorts. Here we quantify plasma NT1 in a large, well‐characterized cohort and examine the association between plasma NT1 and cross‐sectional clinical and biomarkers measures.

**Methods:**

Seven hundred and seventeen individuals enrolled in the Alzheimer's Disease Neuroimaging Initiative (ADNI) who have plasma NT1, Aß‐PET, MRI, and clinical (Clinical Dementia Rating; CDR) measures were included in this study (Table 1). NT1 was assessed using Quanterix Simoa HD‐X platform. PET, MRI, clinical, and other plasma measures were derived using previously described procedures in ADNI. Linear regressions were performed to assess the cross‐sectional association of NT1 with clinical and biomarkers measures, after adjusting relevant covariates.

**Results:**

NT1 levels were elevated in cognitively impaired (MCI/AD; CDR>0) relative to CN (CDR=0) individuals (p=0.008, Figure 1A). Specifically, NT1 is elevated in the MCI group (CDR=0.5, MCI vs Aß‐ CN group: p=0.005), but not the AD group (CDR>0.5, AD vs all other groups: p’s >0.206, Figure 1B). NT1 was associated with plasma phosphorylated (p)Tau‐181 (p=1.27x10‐9, Figure 2A) and plasma neurofilament light chain (NfL; p=5.68x10‐6, Figure 2B) but not hippocampal volume (p=0.239).

**Conclusion:**

Plasma NT1 differentiated CN from MCI/AD individuals and was elevated particularly in the early symptomatic phase of disease. Plasma NT1 was associated with plasma markers of tau and neurodegeneration. Together these results suggest that plasma NT1 may be a useful biomarker of AD‐related tau pathology and neurodegeneration.